# Systemic Effects of Arctic Pollutants in Beluga Whales Indicated by CYP1A1 Expression

**DOI:** 10.1289/ehp.7664

**Published:** 2005-07-14

**Authors:** Joanna Y. Wilson, Suzy R. Cooke, Michael J. Moore, Daniel Martineau, Igor Mikaelian, Donald A. Metner, W. Lyle Lockhart, John J. Stegeman

**Affiliations:** 1 Department of Biology, Woods Hole Oceanographic Institution, Woods Hole, Massachusetts, USA; 2 Department of Veterinary Medicine, University of Pennsylvania, Philadelphia, Pennsylvania, USA; 3 Department of Pathology and Veterinary Microbiology, University of Montréal, Montréal, Québec, Canada; 4 Freshwater Institute, Winnipeg, Manitoba, Canada

**Keywords:** Arctic, beluga whale, CYP1A1, cytochrome P450 1A1, immunohistochemistry, St. Lawrence estuary

## Abstract

Cytochrome P450 1A1 (CYP1A1) is induced by exposure to polycyclic aromatic hydrocarbons (PAHs) and planar halogenated aromatic hydrocarbons (PHAHs) such as non-*ortho* polychlorinated biphenyls (PCBs). In this study, we examined CYP1A1 protein expression immunohistochemically in multiple organs of beluga whales from two locations in the Arctic and from the St. Lawrence estuary. These beluga populations have some of the lowest (Arctic sites) and highest (St. Lawrence estuary) concentrations of PCBs in blubber of all cetaceans. Samples from these populations might be expected to have different contaminant-induced responses, reflecting their different exposure histories. The pattern and extent of CYP1A1 staining in whales from all three locations were similar to those seen in animal models in which CYP1A has been highly induced, indicating a high-level expression in these whales. CYP1A1 induction has been related to toxic effects of PHAHs or PAHs in some species. In St. Lawrence beluga, the high level of CYP1A1 expression coupled with high levels of contaminants (including CYP1A1 substrates, e.g., PAH procarcinogens potentially activated by CYP1A1) indicates that CYP1A1 could be involved in the development of neoplastic lesions seen in the St. Lawrence beluga population. The systemic high-level expression of CYP1A1 in Arctic beluga suggests that effects of PAHs or PHAHs may be expected in Arctic populations, as well. The high-level expression of CYP1A1 in the Arctic beluga suggests that this species is highly sensitive to CYP1A1 induction by aryl hydrocarbon receptor agonists.

Chemical contaminants are ubiquitous in the world’s oceans. Specific health effects and overt disease in fish have been linked to high concentrations of contaminants in some coastal regions of North America (e.g., Murchelano and Wolke 1991; Myers et al. 1990). Atmospheric processes distribute many of these same contaminants to the polar regions (MacDonald et al. 2000), where they accumulate in the fatty tissue of top predators. In the Arctic, prominent contaminants fall into five broad categories: chlorinated industrial compounds including polychlorinated biphenyls (PCBs), organic pesticides, polycyclic aromatic hydrocarbons (PAHs), metals, and radionuclides (MacDonald et al. 2000). Persistent contaminants that accumulate in the lipid-rich blubber of whales include PCBs, DDT (dichlorodiphenyl-trichloroethane), and other chlorinated pesticides. Concentrations of these contaminants in Arctic ecosystems have declined somewhat over the past 20 years, yet they persist in marine mammal species, including the beluga (Muir et al. 1999). Although cetaceans in the Arctic have contaminant concentrations that are at least 10 times lower than the most highly contaminated cetaceans from other locations (Norstrom and Muir 1994), Arctic animals may yet be at risk for adverse health effects.

We obtained multiple internal organ tissue samples from beluga whales that stranded dead along the St. Lawrence estuary and during subsistence hunts in the Mackenzie Delta and Hudson Bay. The St. Lawrence beluga population was designated endangered in 1980 (Seargent 1986), a status continued under the Species at Risk Act in Canada (2002); thus, stranded animals represent the only means to obtain tissues for scientific study from this location. The Mackenzie Delta and Hudson Bay sites harbor two separate Arctic populations of beluga, both of which have levels of PCB and PAH contaminants an order of magnitude lower than those at the St. Lawrence estuary site. These populations, including the two Arctic populations, are geographically separated and represent separate stocks of this species. Beluga are located in offshore waters during winter months and in coastal regions during summer. They have a complex diet including species of fish and crustaceans, which is their primary route of exposure to environmental contaminants.

Among marine mammals, odontocetes (toothed whales), including beluga, may be at the greatest risk of contaminant effects because these animals are top predators that accumulate contaminants to a higher degree than do mysticetes (baleen whales) (O’Shea and Brownell 1994). Concentrations of PCBs as high as 300 μg/g (lipid weight basis) have been recorded in odontocete blubber (Ross et al. 2000). Planar halogenated aromatic hydrocarbons (PHAHs), the dioxin-like contaminants that include non-*ortho* and mono-*ortho* substituted PCBs, are of special concern because even at low doses they can affect development of the immune, nervous, and reproductive systems in animal models (Birnbaum and Tuomisto 2000).

Assessing health effects of contaminants in cetaceans is difficult because experimental exposures are precluded and fresh tissues are rarely available. Molecular changes associated with exposure to selected compounds can suggest whether systemic effects are likely. Cytochrome P450 1A (CYP1A) induction is elicited by PAHs and PHAHs via binding to the aryl hydrocarbon receptor (AHR) (Whitlock 1999). The mammalian CYP1A gene subfamily contains two members: CYP1A1 and CYP1A2. Typically in mammals, CYP1A2 expression is limited to liver, whereas CYP1A1 is more strongly inducible in extrahepatic organs as well as liver. CYP1A1 induction has been correlated to higher-order toxic effects, including thymic atrophy, weight loss, and lethal toxicity induced by PCB, polychlorinated dibenzodioxin, and polychlorinated dibenzofuran exposure in rodents (Safe 1987). Thus, systemic CYP1A1 expression can indicate a risk for toxic effects.

In an earlier study, CYP1A1 levels in liver of Arctic beluga were strongly correlated with the concentration of non-*ortho* and mono-*ortho* PCBs in blubber—compounds that are known to induce CYP1A1 through the AHR (White et al. 1994)—indicating that CYP1A1 is a good biomarker of exposure in beluga. In this study, we examined cellular location and relative levels of CYP1A1 expression in multiple organs of beluga whale from the St. Lawrence estuary and from two locations in the Arctic (Beaufort Sea and western Hudson Bay populations). Samples from these populations might be expected to have different contaminant-induced responses, reflecting their different exposure histories. Beluga from the St. Lawrence estuary population have high concentrations of PCBs, chlorinated pesticides (Muir et al. 1996), and metals (mercury, lead, and selenium) (Wagemann et al. 1990) in blubber. These animals also show highly elevated prevalences of overt pathologies linked to toxicants (Martineau et al. 1988); thus, we addressed the question of whether CYP1A1 might be expressed in multiple organs of beluga from several regions, because this would indicate whether biochemical effects in these animals might be occurring systemically and could indicate the sensitivity of this species to PHAH toxicity.

## Materials and Methods

We obtained tissue samples from multiple organs of beluga whale from three separate populations. The public and officials of various government agencies reported beluga whales found dead, stranded on the St. Lawrence estuary shoreline. The carcasses were immediately transported by truck to the postmortem room of the Faculté de Médecine Vétérinaire of Université de Montréal, 500 km to the southwest, where pathologists assisted by veterinary students examined them upon arrival. Fourteen beluga were included in this study from the St. Lawrence estuary. Samples were additionally collected during subsistence hunts of the Mackenzie Delta (*n* = 15) and Hudson Bay (*n* = 12) beluga populations. The ages of the animals from each population included in this study are shown in Table 1. Standard body measurements, total body, organ, and blubber weights, and tooth counts were determined when possible. Organs sampled included adrenal gland, brain, bladder, colon, gonad (ovary and testis), heart, kidney, liver, lung, skin, and thyroid. The time to necropsy was < 12 hr postmortem for Arctic animals. For the St. Lawrence beluga, carcasses were recovered < 3 days after death judging by the extent of postmortem changes and accounting for the cold water temperature, which retards autolysis. Marked autolysis was seen in the livers of some of the St. Lawrence beluga. Age was determined in the Hudson Bay and St. Lawrence beluga by sectioning teeth longitudinally and counting dentine growth layers on sections using a binocular microscope, using the standard of two growth layers per year (Brodie 1982). We calculated age for the Mackenzie Delta beluga using length measurements and established age–length relationships (Doidge 1990).

The tissues were removed at necropsy, and small samples were fixed in 10% neutral buffered formalin, embedded in paraffin, and sectioned at 5 μm. We assessed CYP1A1 expression by immunohistochemical analysis with monoclonal antibody (Mab) 1-12-3, as previously described (Smolowitz et al. 1991). Mab 1-12-3 recognizes an epitope that in mammals is specific to CYP1A1 but not to CYP1A2 (Drahushuk et al. 1998), and use in Western blot shows a single band in beluga whale liver microsomes (White et al. 1994). We calculated a semiquantitative index (0–15) of CYP1A1 expression determined by immunohistochemistry by multiplying the intensity (0–5) and occurrence (0–3) of label for each cell type in a given organ. A linear relationship between this index and CYP1A protein content measured by immunoblot has been previously shown for expression in liver and for CYP1A1 induced in cells in culture (Hahn et al. 1993; Woodin et al. 1997). Serial sections were stained with the nonspecific antibody UPC-10 (Sigma-Aldrich Co., St. Louis, MO, USA) to control for any nonspecific staining. Replicate slides were stained with hematoxylin and eosin. Although tissue fixation times were not controlled between the samples, they were processed into paraffin blocks within 2 months for all organs except the adrenal and thyroid gland from the St. Lawrence beluga. Epitope recognition with this antibody was equivalent among scup liver samples held in formalin between 2 weeks and 5 months (Smolowitz R, Stegeman J, unpublished observations), a period encompassing the times that beluga tissues were in formalin.

Differences in CYP1A1 expression in the three populations of beluga were determined using analysis of variance and either the Tukey-Kramer or Sheffe’s model when there were unequal or equal numbers of samples, respectively. Samples were always divided between site and sex for statistical analyses.

## Results and Discussion

Analysis of liver and extrahepatic organs showed patterns of CYP1A1 expression consistent with a strong induction of CYP1A1, based on what has been seen in mammalian and non-mammalian vertebrate models [Tables 1 and 2, and supplementary material (http://ehp.niehs.nih.gov/docs/2005/7664/supplement.pdf)]. CYP1A1 expression was seen in vascular endothelial cells in multiple organs of all individuals included in this study, including all lung (*n* = 33) and skin samples (*n* = 13) and nearly all bladder (16 of 18), testes (13 of 17), and adrenal (12 of 13) samples (Table 3). The expression of endothelial CYP1A1 in multiple organs examined from each individual whale indicates a systemic effect of contaminants in the Arctic beluga whale. The levels and patterns of CYP1A1 expression in selected organs are considered below.

### CYP1A1 expression in liver.

**Figure 1:**
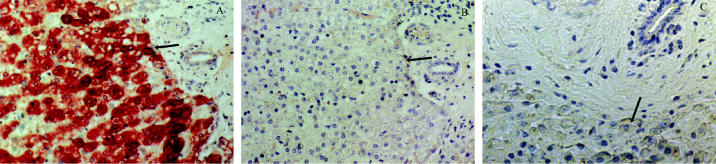
CYP1A1 expression in liver from beluga whale. CYP1A1 is labeled pink to dark red; arrows indicate cells with labeling and the identical cell type without labeling. (*A*) CYP1A1 expression in hepatic parenchyma from an Arctic beluga. (*B*) Serial section from Arctic beluga shown in (*A*) stained using the nonspecific antibody UPC-10. (*C*) CYP1A1 expression in hepatic parenchyma from a St. Lawrence beluga. Magnification, 400×.

CYP1A1 was highly expressed in hepatic parenchyma of Arctic beluga liver (Table 2). Typically in mammalian liver, CYP1A1 expression is localized to periportal parenchyma in untreated or slightly induced animals, and panlobular expression is seen only in animals in which CYP1A1 is strongly induced (Oinonen and Lindros 1998). High-level CYP1A1 expression that is panlobular, as in Figure 1, is fully consistent with CYP1A1 having been strongly induced in liver of Arctic beluga. Surprisingly, the levels of CYP1A1 expression in liver from the highly contaminated St. Lawrence beluga were significantly lower than those in the Arctic animals (Table 2), despite the greater exposure to inducing compounds: liver PCB concentrations in male beluga average 1,445 ng/g and 132 ng/g in the St. Lawrence and Arctic, respectively (Metcalfe et al. 1999). CYP1A1 expression may be suppressed in St. Lawrence beluga liver, potentially as a result of high-level contaminant exposure. CYP1A is suppressed in liver but not in other organs of fish experimentally exposed to high doses of non-*ortho* PCB congeners (e.g., PCB-126) (Schlezinger and Stegeman 2001). Unlike PCBs, high levels of PAHs are not known to suppress CYP1A1 expression.

The time from death to necropsy was longer for the St. Lawrence beluga than for the Arctic beluga, and histologic analyses do show autolysis in these liver samples. Although CYP1A1 expression in other organs was not significantly lower in the St. Lawrence beluga than in Arctic animals, the liver degrades at a faster rate. It is likely that differences in hepatic CYP1A1 expression simply reflect degradation and the difference between time of death and fixation for samples collected from subsistence hunts and strandings. Unfortunately, it is impossible to collect from these sites under identical conditions. There are no subsistence hunts of the St. Lawrence beluga population, and locating stranded animals in the Arctic is not feasible. Yet the pattern and levels of CYP1A1 expression in Arctic animals indicate a substantial induction in those beluga.

### CYP1A1 expression in lung.

**Figure 2:**
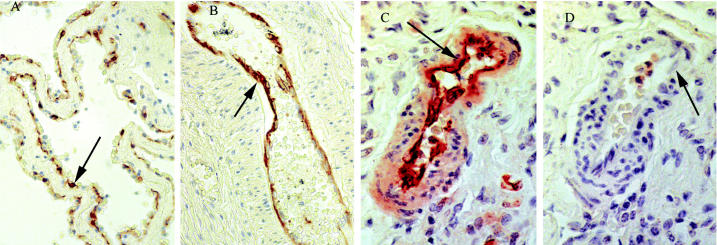
CYP1A1 expression in lung and bladder endothelium from beluga whale. CYP1A1 is labeled pink to dark red; arrows indicate cells with labeling and the identical cell type without labeling. (*A*) CYP1A1 expression in lung endothelium from an Arctic beluga. (*B*) CYP1A1 expression in bladder endothelium from an arteriole from an Arctic beluga. (*C*) CYP1A1 expression in lung endothelium from an Arctic beluga. (*D*) Serial section from Arctic beluga shown in (*C*) was labeled using the nonspecific antibody UPC-10. Magnification: *A*, *C*, *D*, 400×; *B*, 200×.

As indicated above, endothelial CYP1A1 levels (Table 3, Figure 2A,C,D) were high in lung. CYP1A1 expression was seen also in chondrocytes and bronchiolar epithelium but was not seen in type 1 or type 2 pneumocytes [see supplementary material (http://ehp.niehs.nih.gov/docs/2005/7664/supplement.pdf)]. The predominant environmental exposure route for CYP1A1 inducers is dietary, but a recent study in mice suggests that PCB uptake can be greater via inhalation than from diet (Casey et al. 1999). Thus, consideration of nondietary exposures such as inhalation may be warranted in regions where PAH and/or PCB exposure levels are likely to be high. Hormonal, histo-pathologic, and behavioral changes were seen in mice exposed to 0.9 μg/m^3^ Aroclor 1242 in the air (Casey et al. 1999), a concentration that is approximately 10,000-fold higher than Arctic atmospheric PCB concentrations (MacDonald et al. 2000). Atmospheric sources of PCBs in the Arctic could result in an estimated lung exposure of 1.3–67 ng/day in Arctic beluga (Table 4). Likewise, an inhaled PAH exposure could be expected to range from 5.6 to 363 ng/day in Arctic beluga (Table 4), although this exposure would be dominated by lower-molecular-weight PAHs such as fluorene and phenanthrene (MacDonald et al. 2000), which do not typically induce CYP1A (Bols et al. 1999). Given that, and considering that type 1 pneumocytes (the pulmonary cell type primarily involved in gas exchange in the lung) did not express CYP1A1 in beluga, it is more plausible to conclude that CYP1A1 induction in lung was solely the result of dietary exposure and that the contribution of inhaled contaminants was marginal.

### CYP1A1 expression in bladder.

**Figure 3:**
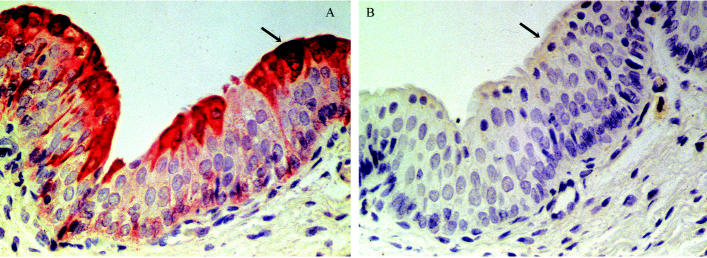
CYP1A1 expression in transitional epithelium from the bladder of Arctic beluga whale. CYP1A1 is labeled pink to dark red; arrows indicate cells with labeling and the identical cell type without labeling. (*A*) CYP1A1 expression in the transitional epithelium of bladder; labeling is most intense in the umbrella cells. (*B*) Serial section from beluga shown in (*A*) labeled using the nonspecific antibody UPC-10. Magnification, 400×.

In bladder, CYP1A1 was highly expressed in both endothelium (Table 3, Figure 2B) and transitional epithelium forming the bladder mucosa (Table 2, Figure 3). CYP1A1 in transitional epithelium was most highly expressed in umbrella cells, the cells in direct contact with urine. A transitional cell carcinoma of the bladder has been found in a beluga from the highly contaminated St. Lawrence estuary (Martineau et al. 1985). In humans, CYP1A is involved in the activation of a variety of potential bladder carcinogens (Gonzalez and Gelboin 1994), is expressed in primary transitional cell tumors of the urinary bladder, and has been correlated with tumor grade (G1–G3) (Murray et al. 1995). CYP1A1 could be involved in the development of bladder tumor in the St. Lawrence beluga population.

The expression of CYP1A1 in bladder was as high in the Arctic beluga as in the St. Lawrence beluga. Considering that, in the transitional epithelium, the most highly induced cells were in direct contact with urine (Figure 3), the induction of CYP1A1 in bladder presumably was caused by contaminants excreted into the urine. Potential CYP1A1 inducers in urine include both PCBs and PAHs. PAHs are eliminated more rapidly and therefore accumulate to a much lower degree than do PCBs, yet higher levels of exposure would still result in significant CYP1A1 induction. In the St. Lawrence estuary, PAH exposures are higher and likely more important for CYP1A1 induction than in the Arctic: Sediment-associated PAHs are 500–4,500 ng/g (Martel et al. 1986) and 400–980 ng/g (MacDonald et al. 2000) in the St. Lawrence and Arctic, respectively. The relative contribution of PCBs and PAHs to urinary contaminants is unknown in the St. Lawrence beluga. In the Arctic, where contaminants are atmospherically derived, atmospheric PAHs are dominated by those that do not induce CYP1A1 (MacDonald et al. 2000); thus, PAH contributions to CYP1A1 induction may be minimal in Arctic beluga whales.

PCBs that are AHR agonists are highly correlated to CYP1A1 levels in liver of Arctic beluga (White et al. 1994), indicating that induction of CYP1A1 in bladder of Arctic beluga is likely to be related to PCB exposure. In mice exposed to PCBs in the diet, only 5% of oral dose appeared in urine, mainly as conjugates (Wehler et al. 1989). We calculated a total body burden based on PCB concentrations in the blubber, blubber weight, and total body weight (Table 5). Given this total body dose, we can estimate an upper limit on the maximal oral dose in Arctic beluga. The urinary PCB concentrations would be very small in the Arctic beluga, presumably much less than 0.05–0.1 mg/kg (5% of the upper limit on the maximal oral dose), because we would expect most urinary PCBs to be conjugated and not able to induce CYP1A1. These results suggest that the doses required for CYP1A1 induction in bladder are low and indicate that beluga are very sensitive in their responses to PHAH contaminants. Determining urinary concentrations of PAHs and PCBs would be important to confirm that such compounds are present in this organ and what concentrations are responsible for such high-level CYP1A1 expression.

### CYP1A1 expression in testis.

Moderate levels of CYP1A1 expression were seen in the spermatogenic series in the testis (Table 1). Although this may have included some Sertoli cells, CYP1A1 expression appeared primarily in spermatogonia and spermatocytes. In studies with several other mammals, testicular microsomal preparations have shown very low or no CYP1A activity (Machala et al. 1998; Revel et al. 2001; Roman et al. 1998). Testicular CYP1A activity was not induced in rats (Roman et al. 1998), bulls (Machala et al. 1998), or mice (Revel et al. 2001) exposed to a variety of inducers, although the AHR and dimerization partner ARNT (aryl hydrocarbon receptor nuclear translocator), which are required for CYP1A induction, are present in testis (Roman et al. 1998). Immunohistochemical analyses of mouse testes showed CYP1A1 in interstitial cells only, and this was reportedly not induced by benzo[*a*]pyrene (Revel et al. 2001). CYP1A1 expression in the spermatogenic series is an unusual finding in a mammalian species. Considering that CYP1A1 is involved in the activation of procarcinogens and generation of reactive oxygen species, high-level CYP1A1 expression in the spermatogenic series could be significant for sperm function and gamete development.

### Implications of CYP1A1 induction in beluga.

The high levels of CYP1A1 expression in the beluga whale from both the Arctic and the St. Lawrence estuary are consistent with high sensitivity of this species to CYP1A inducers. Interestingly, except for the liver, the level of expression was not markedly different between animals from the St. Lawrence estuary and those from the Arctic, despite significant differences in contaminant exposure and apparent tumor prevalence. No tumors were found in 50 Arctic beluga examined, whereas 21 tumors were found in 100 St. Lawrence beluga, resulting in an annual cancer rate of 163 per 100,000 animals calculated for the St. Lawrence estuary beluga (Martineau et al. 2002). However, the necropsies on the Arctic beluga were not as detailed as necropsies on the St. Lawrence animals, and the Arctic animals examined were much younger. Detailed necropsies will need to be performed on older Arctic animals to determine the prevalence of tumors in Arctic populations of beluga.

Liver CYP1A1 expression was previously shown to be highly correlated to mono-*ortho* and non-*ortho* PCBs, ligands for the AHR, in the blubber of Arctic beluga whale (White et al. 1994). The high levels and cellular patterns of CYP1A1 expression are similar to those seen in animal models exposed to high levels of inducers; other mammals do not show this broad pattern of induction unless exposed to high concentrations of contaminants. Thus, even lower doses of contaminants, like those seen in the Arctic animals, appear able to highly induce CYP1A1 in beluga. Beluga whales express levels of CYP1A1 in various organs that are similar to or greater than levels of expression in organs of other cetaceans for which such data are available (Wilson 2003), despite having lower levels of potential inducers in their tissues. These data support the idea that beluga are a more sensitive species, at least compared with other cetaceans. The sensitivity of beluga to CYP1A1 inducers is reflected also in the beluga AHR. The beluga AHR has been cloned, expressed, and shown to bind 2,3,7,8-tetrachlorodibenzo-*p*-dioxin (a prototypical inducer) with a similar binding affinity to that of the C57 strain mouse (Jensen and Hahn 2001). This strain of mouse is highly sensitive to PHAH toxicity (Shen et al. 1991), and the AHR properties suggest that beluga may be similarly sensitive to these contaminants. In the St. Lawrence estuary, only beluga, and not other resident cetacean species, have been found with tumors (De Guise et al. 1994), indicating that beluga may also be particularly susceptible to chemical carcinogenesis. The fact that beluga show a systemic response to CYP1A1 inducers, even at lower doses, indicates that other toxic effects elicited by AHR agonists may be expected, even in populations from the relatively uncontaminated Arctic.

## Conclusions

Although Arctic cetaceans have contaminant concentrations that are among the lowest reported, these exposures still could be biologically significant. Despite such low exposures, Arctic beluga have a pattern and extent of CYP1A1 expression that is similar to those seen in animal models that are maximally induced. These data, supported by *in vitro* studies of the beluga AHR (Jensen and Hahn 2001), and the presence of tumors only in beluga but not other cetaceans resident in the St. Lawrence estuary (De Guise et al. 1994), suggest that beluga are highly sensitive to CYP1A1 inducer substrates. In the St. Lawrence estuary, CYP1A1 could be involved in the development of neoplastic lesions seen in this beluga population (Martineau et al. 1994). Because beluga have a systemic response to PHAH contaminants at low doses, toxic effects may be expected in Arctic populations.

## Supplementary Material

Supplemental Material

## Figures and Tables

**Table 1:** Sample summary.

Site	No.	Age (years)	Published PCB concentrations (μg/g)^a^
Mackenzie Delta	12 Male	> 9^b^	4.9
	3 Female	4–5^b^	—
Hudson Bay	9 Male	4.5–13	2.7
	3 Female	7–17.5	—
St. Lawrence	7 Male	Neonate to 26	78.9
	7 Female	5–31.5	29.6

—, data not available.

a PCB concentrations were determined in blubber and are based on published data (Muir et al. 1996) and not from samples included in this study.

b Based on age–length relationships (Doidge 1990).

**Table 2:** CYP1A1 expression in epithelia of selected internal organs of beluga whale determined immunohistochemically.^a^

Site	Sex	Liver hepatic parenchyma	Bladder transitional epithelium	Testis spermatogenic series^b^
Mackenzie Delta	Male	11.7 ± 2 (12)	8 ± 1.2 (7)	6.3 ± 1.5 (12)
	Female	10 ± 2.8 (2)	—	—
Hudson Bay	Male	12.4 ± 2.1 (7)	—	3 ± 1.4 (2)
	Female	10 ± 3.5 (3)	12 (1)	—
St. Lawrence	Male	0.3 ± 0.8 (8)*	6 ± 8.5 (6)	4.5 ± 1.2 (4)
	Female	3.3 ± 3.2 (6)*	11.25 ± 2.9 (4)	—

—, organ not available.

a CYP1A1 expression levels shown are means ± SD (*n*). CYP1A1 expression is on a scale of 0–15, based on occurrence and intensity of staining, as described in “Materials and Methods.”

b May include some Sertoli cells.

* Mean is significantly different than other sites at *p* < 0.05.

**Table 3:** Endothelial CYP1A1 expression in selected internal organs of beluga whale determined immunohistochemically.^a^

Site	Sex	Brain	Bladder	Gonad	Kidney	Liver	Lung
Mackenzie Delta	M	1.6 ± 1.4 (12)	8 ± 1.2 (7)	5 ± 2.6 (12)	6.5 ± 2.1* (12)	3.75 ± 3.1 (12)	9.1 ± 2.1 (12)
	F	1.5 ± 2.1 (2)	—	2.25 ± 3.2 (2)	9 ± 1.4* (2)	2 ± 2.8 (2)	8 ± 2.8 (2)
Hudson Bay	M	1.7 ± 2.6 (6)	—	1 ± 1.4 (2)	1.8 ± 2.3 (9)	2.8 ± 4.1 (7)	6.7 ± 3.7 (9)
	F	0 ± 0 (2)	0 (1)	—	0 ± 0 (3)	0.7 ± 1.2 (3)	5.5 ± 1.8 (3)
St. Lawrence	M	2.3 ± 3.7 (8)	7.6 ± 6.1 (6)	2.7 ± 4.6 (4)	0 ± 0 (7)	0 ± 0 (8)	7 ± 1.4 (4)
	F	3 ± 3.0 (5)	13.1 ± 3.8 (4)	1.25 ± 1.5 (4)	2.2 ± 2.8 (7)	1.6 ± 1.9 (6)	6.3 ± 3.5 (5)

Abbreviations: —, organ not available; F, female; M, male.

a CYP1A1 expression levels shown are means ± SD (*n*). CYP1A1 expression is on a scale of 0–15, based on occurrence and intensity of staining, as described in “Materials and Methods.”

* Mean is significantly different than other sites at *p* < 0.05.

**Table 4:** PCB and PAH dose to lungs of Arctic beluga whale via inhalation.

Parameter	Range of value
Tidal volume (L)	
Bottlenose dolphin^a^	10
Gray whale^a^	62
Respiration rate (breaths/min)	
Bottlenose dolphin^b^	2
Weddell seal^a^	8
Volume inspired^c^	0.29–7.1 × 10^5^ L/day
Air concentrations (pg/m)^d^	
PCBs	44–94.3
PAHs	194–508
Lung dose (ng/day)^e^	
PCBs	1.3–67
PAHs	5.6–363

a Wartzok (2002).

b Cockcroft and Ross (1990).

c Tidal volume (L) × respiration rate (breaths/min) × 1,440 min/day.

d MacDonald et al. (2000).

e Air concentration (pg/m) × volume inspired (L/day) × 0.001 m^3^/L.

**Table 5:** Whole-body dose of PCBs in Arctic beluga whale in this study.

Parameter	Range of value
Length	335–447 cm
Body weight^a^	474–995 kg
Blubber weight^b^	189.6–398 kg
Blubber contaminants^c^	2.7–4.9 μg/g
Whole-body dose^d^	1.08–1.96 mg/kg

a Weight (kg) = 10^−3.84^ length (cm)^2.58^ (Doidge 1990).

b Body weight (kg) × percent body weight as blubber; blubber weight is 40% body weight in beluga whale (Seargent and Brodie 1969).

c Muir et al. (1996).

d Blubber contaminants (mg/kg) × blubber weight (kg) ÷ body weight (kg).
