# Supplementary material for: Systemic Effects of Arctic Pollutants in Beluga Whales Indicated by CYP1A1 Expression
Source: Environ Health Perspect. 2005 Jul 14;113(11):1594–9. doi: 10.1289/ehp.7664 (PMC1310924; doi:10.1289/ehp.7664)
Supplement: Supplemental Material [file ehp0113-001594s1.pdf]

Supplemental Data, Table 1. CYP1A1 Expression in Internal Organs from Beluga Whale from the Arctic (Mackenzie Delta and Hudson Bay) and the St. Lawrence Estuary. Data shown was collected by immunohistochemistry, data is shown only for cell types that stained for CYP1A1 expression with the monoclonal antibody 1-12-3 in at least one individual. CYP1A1 expression is scored on a scale of 0-15. Data shown is mean expression ± standard deviation (N) for males (M) and females (F) from each location.

Organ	Cell Type	Mackenzie Delta		Hudson Bay		St. Lawrence Estuary	
		M	F	M	F	M	F
Heart	Endothelia	-	-	1.3 ±1.7 (9)	3.0 ±0 (3)	-	-
Heart	Epicardium	-	-	0.8 ±1.7 (9)	0.0 (1)	-	-
Cerebrum	Endothelia	1.6 ±1.4 (12)	1.5 ±2.1 (2)	1.7 ±2.6 (6)	0.0 ±0 (2)	2.3 ±3.7 (8)	3.0 ±3.0 (5)
Cerebrum	Vascular Smooth Muscle	4.7 ±1.6 (12)	1.5 ±1.7 (2)	0.0 ±0 (6)	0.0 ±0 (2)	0.0 ±0 (8)	0.0 ±0 (5)
Cerebrum	Neuron	4.8 ±1.9 (12)	3.5 ±0.7 (2)	0.0 ±0 (6)	0.0 ±0 (2)	0.0 ±0 (8)	0.2 ±0.4 (5)
Cerebrum	Neuroglia	3.0 ±1.5 (12)	2.0 ±1.4 (2)	0.8 ±2 (6)	0.0 ±0 (2)	0.8 ±1.8 (8)	2.2 ±2.5 (5)
Skin[1]	Endothelia	5.5 ±1.5 (11)	3.5 ±0.7 (2)	-	-	-	-
Skin	Vascular Smooth Muscle	3.8 ±0.6 (11)	3.0 ±1.4 (2)	-	-	-	-
Skin	Peripheral Nerve	2 (1)	*	-	-	-	-
Lung	Endothelia	9.1 ±2.1 (12)	8.0 ±2.8 (2)	6.7 ±3.7 (9)	5.5 ±1.8 (3)	7.0 ±1.4 (4)	6.3 ±3.5 (5)
Lung	Vascular Smooth Muscle	5.6 ±1.1 (12)	4.0 ±0 (2)	0.0 ±0 (9)	0.0 ±0 (3)	0.0 ±0 (4)	0.0 ±0 (5)
Lung	Bronchial Epithelia	4.0 (1)	*	0.5 ±1.5 (9)	0.0 ±0 (3)	0.0 ±0 (4)	0.0 ±0 (5)
Lung	Chondrocytes	3.5 ±1.9 (12)	0.5 ±0.7 (2)	0.6 ±1.1 (9)	0.0 ±0 (3)	0.0 ±0 (4)	0.0 ±0 (5)

Colon	Endothelia	5.6 ±2.6 (5)	-	-	-	-	-
Colon	Muscularis Mucosae	6.3 ±2 (5)	-	-	-	-	-
Colon	Goblet Cells	0.4 ±0.9 (5)	-	-	-	-	-
Colon	Absorptive Cells	0.6 ±1.3 (5)	-	-	-	-	-
Colon	Peripheral Nerve	5 ±1.2 (4)					
Liver	Endothelia	3.8 ±3.1 (12)	2.0 ±2.8 (2)	2.9 ±4.1 (7)	0.7 ±1.2 (3)	0.0 ±0 (8)	1.6 ±1.9 (6)
Liver	Hepatic parenchyma	11.7 ±2 (12)	10.0 ±2.8 (2)	12.4 ±2.1 (7)	10.0 ±3.5 (3)	0.3[4] ±0.8 (8)	3.3[4] ±3.2 (6)
Liver	Bile Collecting Duct Epithelia	0.0 ±0 (12)	0.0 ±0 (2)	0.0 ±0 (7)	1.0 ±1.7(3)	0.5 ±1.2 (8)	1.0 ±2.4 (6)
Kidney	Endothelia	6.5[4] ±2.1 (12)	9.0[4] ±1.4 (2)	1.8 ±2.3 (9)	0.0 ±0 (3)	0.0 ±0 (7)	2.2 ±2.8 (7)
Kidney	Vascular Smooth Muscle	4.9 ±1.3 (12)	4.0 ±0 (2)	0.0 ±0 (9)	0.0 ±0 (3)	0.0 ±0 (7)	0.0 ±0 (7)
Kidney	Renal Corpuscle	1.3 ±1.2 (12)	0.0 ±0 (2)	0.0 ±0 (9)	0.0 ±0 (3)	0.0 ±0 (7)	0.0 ±0 (7)
Kidney	Tubular Epithelia - Cortex	8.9[4] ±0.4 (12)	8.3[4] ±1.1 (2)	0.4 ±1.3 (9)	0.0 ±0 (3)	0.0 ±0 (7)	0.9 ±2.3 (7)
Kidney	Tubular Epithelia - Medulla	3.6 ±1.3 (12)	1.8 ±0.4 (2)	1.2 ±1.8 (9)	1.0 ±0 (3)	0.0 ±0 (7)	0.0 ±0 (7)
Bladder	Endothelia	8.0 ±1.2 (7)	-	-	0.0 (1)	7.6 ±6.1 (6)	13.1 ±3.8 (4)
Bladder	Smooth Muscle of the Bladder	6.3 ±0.9 (7)	-	-	6.0 (1)	6.0 ±2.4 (6)	5.3 ±3.8 (4)
Bladder	Transitional Epithelia	8.0 ±1.2 (7)	-	-	12.0 (1)	6.0 ±8.5 (6)	11.3 ±2.9 (4)
Adrenal gland	Endothelia	7.1 ±2.5 (9)	8.3 ±6 (2)	-	-	-	3.0 ±4.2 (2)

Adrenal gland	Vascular Smooth Muscle	5.7 ±0.8 (9)	2.5 ±0.7 (2)	-	-	-	0.0 ±0 (2)
Adrenal gland	Cortex	6.8 ±1.7 (9)	5.0 ±0 (2)	-	-	-	5.0 ±1.4 (2)
Adrenal gland	Medulla	1.4 ±1.5 (9)	0.0 ±0 (2)	-	-	-	6.5 ±4.9 (2)
Thyroid	Endothelia	-	-	-	-	-	10.5 ±6.4 (2)
Ovary	Endothelia		2.3 ±3.2 (2)		-		1.3 ±1.5 (4)
Ovary	Primordial Follicles		*		-		3.3 ±1.9 (4)
Ovary	Luteal Cells[2]		3.0 ±4.2 (2)		-		*
Ovary	Medulla		*		-		2.5 ±0.6 (4)
Testes	Endothelia	5.0 ±2.6 (12)		1.0 ±1.4 (2)		2.7 ±4.6 (4)	
Testes	Vascular Smooth Muscle	5.6 ±1.7 (12)		0.0 ±0 (2)		0.0 ±0 (4)	
Testes	Spermatogenic Series[3]	6.3 ±1.5 (12)		3.0 ±1.4 (2)		4.7 ±1.2 (4)	
Testes	Leydig Cells	3.3 ±1.5 (12)		1.0 ±1.4 (2)		0.0 ±0 (4)	

- organ not available, * cell type not present in section examined, [1] Skin samples from 3 adult captive beluga showed no CYP1A1 staining in any cell type, [2] corpus luteum of pregnancy, [3] primarily spermatogonia and primary spermatocytes, may include some Sertoli cells, [4] significantly different from animals from other sites at $p<0.05$.

Supplemental Data, Table 2. CYP1A1 Expression in Internal Organs from a Beluga Whale Neonate. Data shown was collected by immunohistochemistry, data is shown only for cell types that stained for CYP1A1 expression with the monoclonal antibody 1-12-3. CYP1A1 expression is scored on a scale of 0-15. This animal died 7 minutes after birth in a captive facility. The animal identification number is 94-457.

Organ	Cell Type	CYP1A1 Expression
Heart	Endothelia	3
Heart	Endothelia of Epicardium	6
Heart	Mesothlial Cells of Epicardium	3
Heart	Leucocytes[2]	8
Cerebrum	Endothelia	4
Cerebrum	Neuron	2
Muscle	Macrophages	3
Lung	Endothelia	8
Forestomach	Endothelia	3
Forestomach	Leucocytes	3
Forestomach	Epithelia	3
Stomach[1]	Endothelia	8
Stomach	Leucocytes	3
Small Intestine	Endothelia	4
Small Intestine	Leucocytes	3
Liver	Endothelia	3
Liver	Hepatic Parenchyma	4
Pancreas	Endothelia	1
Kidney	Endothelia	8
Kidney	Tubular Epithelia - Cortex	6
Adrenal	Leucocytes	6
Adrenal	Endothelia	4
Spleen	No staining in any cell type	-
Thymus	No staining in any cell type	-

[1]The cetacean stomach is a diverticulated composite stomach with 4 chambers. This sample was not taken by the forestomach, but from one of the other chambers. [2] Leucocytes staining for CYP1A1 consisted primarily of macrophages in this animal.
